# Adult-onset juvenile nasopharyngeal angiofibroma with delayed adult recurrence managed by revision endoscopic resection and external carotid branch ligation

**DOI:** 10.1093/jscr/rjag293

**Published:** 2026-04-23

**Authors:** Aditya Pachwa, Laasya Reddy Arimanda Chaitanya, Meghana Narendula, Lakshya Nehal Samineni

**Affiliations:** Mamata Academy of Medical Sciences, Hyderabad 500118, India; Malla Reddy Institute of Medical Sciences, Hyderabad 500055, India; Malla Reddy Institute of Medical Sciences, Hyderabad 500055, India; College of Medical Sciences, Bharatpur 44600, Nepal

**Keywords:** angiofibroma, nasopharyngeal neoplasms, neoplasm recurrence local, endoscopy, carotid artery external

## Abstract

Juvenile nasopharyngeal angiofibroma (JNA) is a histologically benign but highly vascular skull-base tumor classically seen in adolescent males; adult-onset disease is rare and may be overlooked. Reports of JNA presenting in adulthood with recurrence occurring entirely in adulthood remain scarce. We report a 34-year-old man with recurrent left-sided epistaxis and intermittent nasal obstruction three years after endoscopic resection at age 31 for histologically confirmed JNA. Endoscopy demonstrated a soft mass arising from the left sphenopalatine region. CT showed recurrent disease involving the left nasal cavity, maxillary and sphenoid sinuses, with extension into the sphenopalatine/pterygopalatine fossae and bony erosion (Radkowski IIC). Revision endoscopic coblation-assisted excision with posterior maxillary wall drilling was performed. Significant intraoperative bleeding required selective ligation of external carotid branches (internal maxillary, ascending pharyngeal, and facial arteries) to achieve hemostasis. At 6 months, the patient remained symptom-free.

## Introduction

Juvenile nasopharyngeal angiofibroma (JNA) is a histologically benign yet highly vascular fibrovascular tumor arising from the posterior nasal cavity/nasopharynx, most often near the sphenopalatine foramen. It classically affects adolescent males and typically presents with recurrent epistaxis and unilateral nasal obstruction, with characteristic submucosal skull-base extension [1–3]. Diagnosis is primarily clinicoradiological using contrast-enhanced CT and/or MRI to define extent and bony change, and biopsy is generally avoided due to hemorrhage risk [1, 4]. Adult-onset JNA is uncommon, and recurrence occurring entirely in adulthood after an initial adult presentation has been sparsely reported [3]. We report adult-onset JNA at 31 years with symptomatic recurrence at 34 years, managed by revision endoscopic excision with escalation to external carotid system vascular control for hemostasis.

## Case presentation

A 34-year-old male presented with left-sided epistaxis for one week. Bleeding was moderate, spontaneous, aggravated by nose blowing, and self-limited. He reported intermittent left-sided epistaxis over the preceding three years, with associated intermittent left nasal obstruction (worse during bleeding episodes), episodic headache/heaviness, post-nasal drip, halitosis, nasal discharge, and hyponasal speech. There was no history of recurrent upper respiratory infections, throat pain, aural symptoms, mouth breathing, snoring, dyspnea, or medical comorbidities.

Three years earlier (age 31), he had undergone transnasal endoscopic tumor resection with endoscopic medial maxillectomy and coblation-assisted excision for JNA; histopathology confirmed juvenile nasopharyngeal angiofibroma and recovery was uneventful.

**Figure 1:**
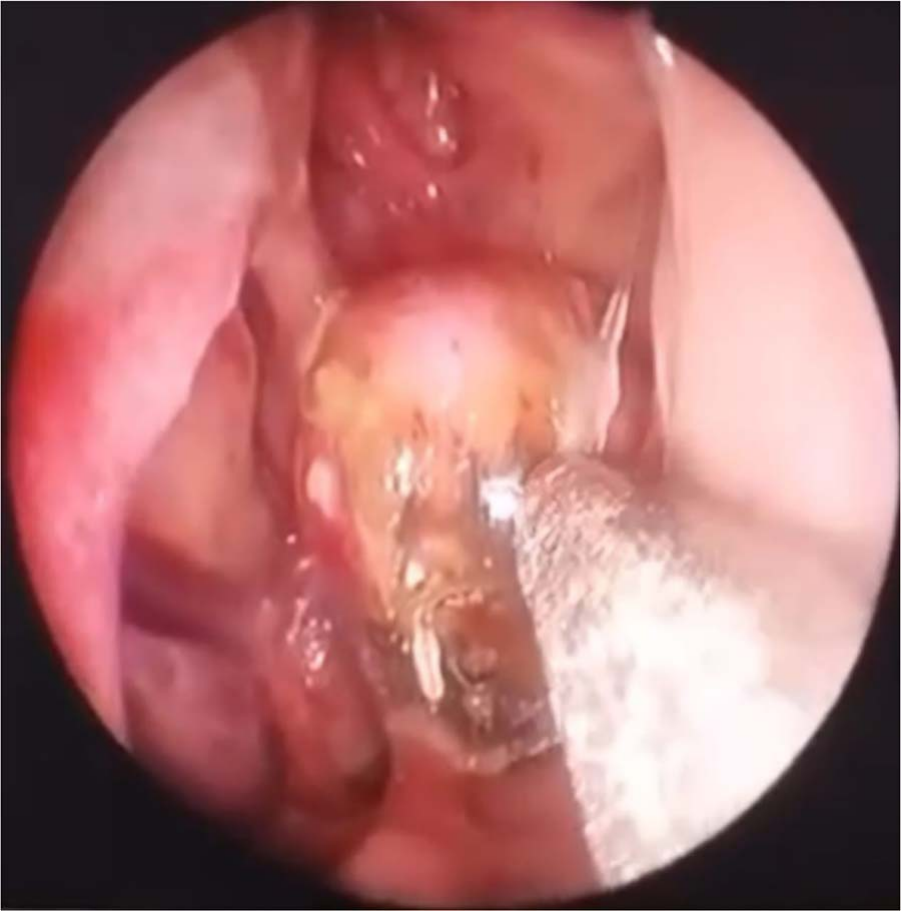
Diagnostic nasal endoscopy (left nasal cavity) smooth, lobulated, hypervascular soft-tissue mass arising from the left sphenopalatine region with extension toward the nasopharynx, consistent with recurrent JNA.

**Figure 2:**
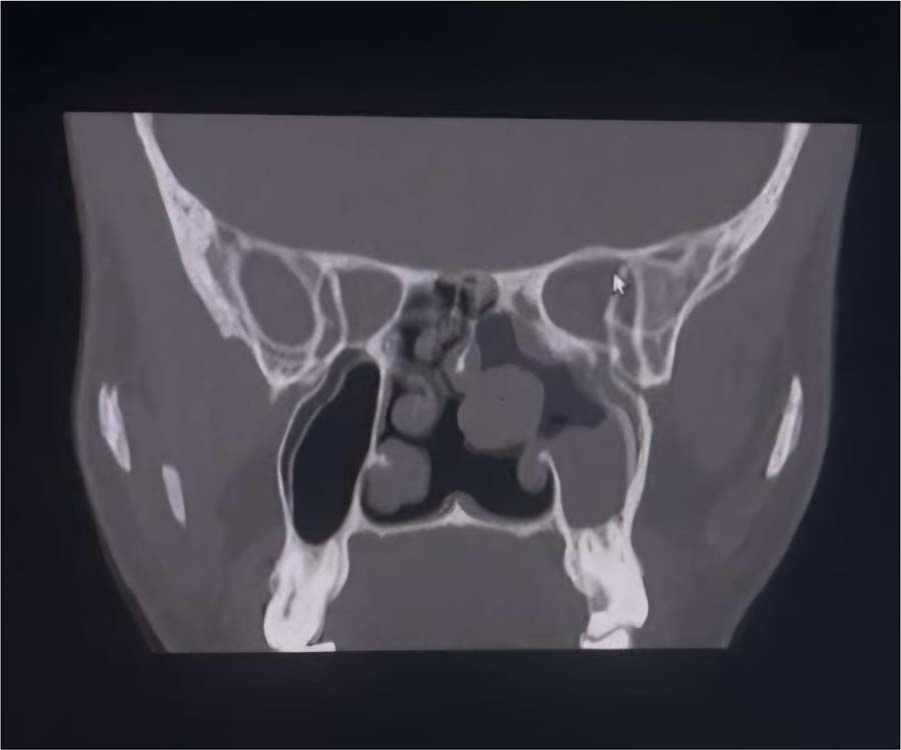
Coronal CT PNS (bone window) soft-tissue mass involving the left nasal cavity with associated left maxillary sinus opacification/extension and sinonasal involvement consistent with recurrent JNA spread.

**Figure 3:**
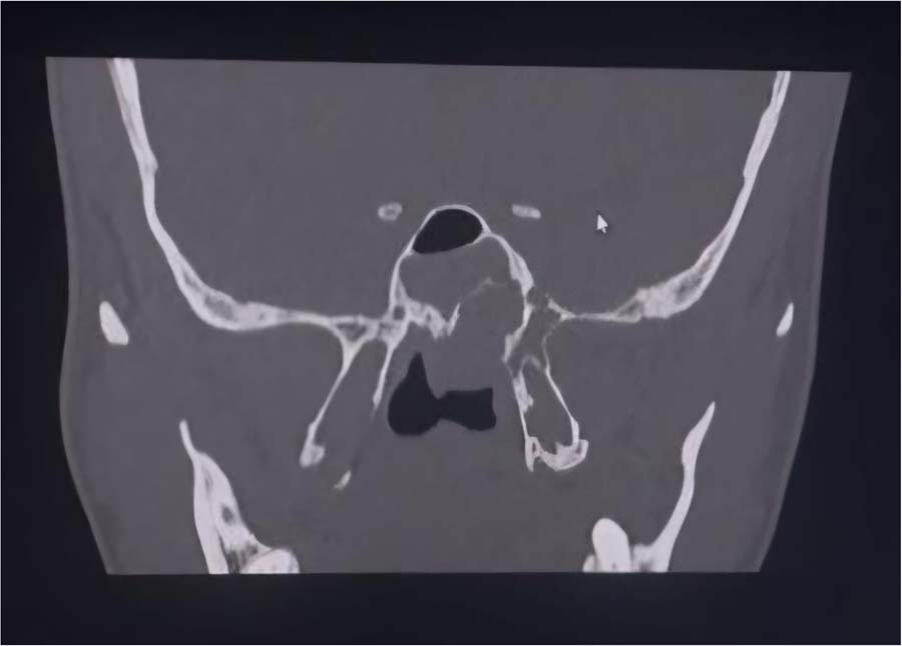
Coronal CT PNS (bone window) recurrent soft-tissue lesion centered at the left sphenopalatine/pterygopalatine region with posterior extension and adjacent bony remodeling erosion.

On current evaluation, external nasal examination was unremarkable. Anterior rhinoscopy showed a deviated nasal septum to the left with a right-sided septal spur. Otologic, facial nerve, and oropharyngeal examinations were normal. Nasal endoscopy revealed postoperative changes and a soft, encapsulated mass arising from the left sphenopalatine region extending into the nasopharynx (Fig. 1). CT of the paranasal sinuses demonstrated a soft-tissue lesion involving the left nasal cavity, left maxillary sinus, and sphenoid sinus with extension into the sphenopalatine and pterygopalatine fossae, with bony erosion of the sphenoid sinus and pterygoid plates—consistent with recurrent JNA (Fig. 2). Based on radiologic extent, the lesion was staged Radkowski IIC (Fig. 3).

The patient underwent revision endoscopic coblation-assisted excision under general anesthesia. The tumor arose from the left sphenopalatine foramen with extension into the sphenoid sinus and pterygomaxillary fossa, with attachment to the posterior nasal septum. Posterior maxillary wall drilling was performed to access deep attachment sites and facilitate complete excision. Significant intraoperative hemorrhage compromised safe endoscopic dissection and necessitated escalation to vascular control through a horizontal cervical incision; the internal maxillary, ascending pharyngeal, and facial arteries were ligated, achieving effective hemostasis (Video 1). Postoperative recovery was uneventful. At 6 months follow-up, he remained symptom-free.

Histopathology showed multiple grey-white to grey-brown firm-to-soft fragments (3.0 × 2.5 × 1.5 cm). Microscopy revealed vascular channels embedded in a fibrous stroma with scattered fibroblasts and no atypia, consistent with JNA.

## Discussion

JNA is histologically benign yet clinically aggressive due to abundant vascularity and characteristic submucosal skull-base spread with bony remodeling/erosion [1]. Adult presentations are distinctly uncommon and may be overlooked because symptoms can mimic inflammatory sinonasal disease or other nasopharyngeal masses [5, 6]. This case is notable because both the index tumor (31 years) and symptomatic recurrence (34 years) occurred entirely in adulthood, reinforcing that JNA should remain in the differential diagnosis for unilateral epistaxis with obstructive symptoms even beyond the typical adolescent age range [1, 5, 6].

Epistaxis and unilateral nasal obstruction remain hallmark symptoms. In suspected recurrence, endoscopy and contrast-enhanced imaging are preferred over biopsy due to the hemorrhage risk associated with tissue sampling [1, 4]. In this patient, imaging demonstrated a sphenopalatine-region origin with sphenoid involvement and deep lateral extension into the pterygopalatine/pterygomaxillary region with bony erosion—features consistent with recognized routes of spread and essential for defining endoscopic corridors and anticipating bleeding risk [1, 2, 7].

Recurrence remains a key challenge in JNA management and is most often related to residual disease in anatomically constrained subsites rather than age alone.

Higher stage, sphenoid invasion, and greater intraoperative bleeding have been associated with increased recurrence risk [8]. These patterns support structured postoperative surveillance, particularly in higher-stage tumors and revision cases.

Surgery remains the mainstay for primary and recurrent JNA [1, 2]. Endoscopic endonasal resection is increasingly favored when feasible; a meta-analysis reported recurrence outcomes that were at least comparable—and in some series lower—than open approaches, likely reflecting improved visualization and access to critical subsites such as the pterygopalatine region and sphenoid recess [8, 9]. Revision surgery is inherently more challenging due to fibrosis, altered landmarks following prior medial maxillectomy, and re-established collateral vascularity, increasing technical difficulty and bleeding risk [2].

Hemorrhage control is pivotal. Preoperative embolization can reduce intraoperative blood loss and operative demands, but it may not be feasible in all settings and may not fully mitigate bleeding in recurrent or laterally extended disease [6, 10]. In this case, embolization was not performed due to patient-related constraints, and escalation to vascular control was required when bleeding compromised safe endoscopic dissection. JNA vascular supply is commonly dominated by the internal maxillary artery with frequent contribution from the ascending pharyngeal and other external carotid branches; recurrent lesions may recruit additional pedicles [1, 4]. Selective external carotid branch ligation, as used here, can be an effective adjunct to achieve hemostasis and permit completion of resection when bleeding limits safe continuation of endoscopic surgery [1, 2, 4]. Minimally invasive endoscopic endonasal techniques have also been reported with favorable outcomes in selected non-embolized cases [11].

## Conclusion

Adult-onset JNA with recurrence occurring entirely in adulthood is rare and may be overlooked in adults presenting with unilateral epistaxis and nasal obstruction. This case underscores that recurrence is driven mainly by tumor extent and residual disease in anatomically constrained subsites, supporting close follow-up in higher-stage and revision settings. Revision endoscopic excision can achieve good outcomes, but surgeons should anticipate altered anatomy and significant hemorrhage and be prepared to escalate hemostasis, including external carotid system vascular control when required. We recommend structured postoperative surveillance with interval MRI for at least 3 years to detect early, potentially asymptomatic recurrence.

## Supplementary Material

Video_rjag293

Video_1_caption_rjag293
