# Supplementary material for: Adult-onset juvenile nasopharyngeal angiofibroma with delayed adult recurrence managed by revision endoscopic resection and external carotid branch ligation
Source: J Surg Case Rep. 2026 Apr 23;2026(4):rjag293. doi: 10.1093/jscr/rjag293 (PMC13105167; doi:10.1093/jscr/rjag293)
Supplement: Video_1_caption_rjag293 [file video_1_caption_rjag293.docx]

Video 1 Intraoperative endoscopic view of revision coblation-assisted excision of recurrent juvenile nasopharyngeal angiofibroma (JNA), demonstrating brisk intraoperative hemorrhage
